# Discharging the medial knee compartment: comparison of pressure distribution and kinematic shifting after implantation of an extra-capsular absorber system (ATLAS) and open-wedge high tibial osteotomy—a biomechanical in vitro analysis

**DOI:** 10.1007/s00402-022-04496-0

**Published:** 2022-06-14

**Authors:** Ferdinand Kloos, Christoph Becher, Benjamin Fleischer, Max Ettinger, Lisa Bode, Hagen Schmal, Andreas Fuchs, Sven Ostermeier, Gerrit Bode

**Affiliations:** 1 Department of Orthopedics and Trauma Surgery, Faculty of Medicine, Clinic of Orthopedic Surgery and Traumatology, Freiburg University Hospital, Medical Center-Albert-Ludwigs-University of Freiburg, Hugstetter Str. 55, 79106 Freiburg, Germany; 2 Department of Orthopedic Surgery, Hannover Medical School, Hanover, Germany; 3 ATOS Klinik Heidelberg, Heidelberg, Germany; 4 University Hospital Odense, Sdr. Boulevard 29, Odense C, 5000 Odense, Denmark; 5 MVZ Gelenk-Klinik Gundelfingen, Gundelfingen, Germany; 6 Sportopaedicum Straubing, Straubing, Germany

**Keywords:** Unicompartmental osteoarthritis, Unloading, Osteotomy, Medial compartment

## Abstract

**Purpose:**

Young and active patients suffering early degenerative changes of the medial compartment with an underlying straight-leg axis do face a therapeutical gap as unloading of the medial compartment cannot be achieved by high tibial osteotomy. Extracapsular absorbing implants were developed to close this existing therapeutical gap. Purpose of the present cadaveric biomechanical study was to compare the unloading effect of the knee joint after implantation of an extra-articular absorber system (ATLAS) in comparison to open-wedge high tibial osteotomy (OW-HTO) under physiological conditions. The hypothesis of the study was that implantation of an extra-capsular absorber results in an unloading effect comparable to the one achievable with OW-HTO.

**Methods:**

Eight fresh-frozen cadaveric knees were tested under isokinetic flexion–extension motions and physiological loading using a biomechanical knee simulator. Tibiofemoral area contact and peak contact pressures were measured using pressure-sensitive film in the untreated medial compartment. The tibiofemoral superior–inferior, latero-medial translation and varus/valgus rotation were measured with a 3D tracking system Polaris. Pressures and kinematics changes were measured after native testing, ATLAS System implantation and OW-HTO (5° and 10° correction angles) performed with an angular stable internal fixator (TomoFix).

**Results:**

The absorber device decreased the pressure in the medial compartment near full extension moments. Implantation of the ATLAS absorbing system according to the manufacturers’ instruction did not result in a significant unloading effect. Deviating from the surgery manual provided by the manufacturer the implantation of a larger spring size while applying varus stress before releasing the absorber resulted in a significant pressure diminution. Contact pressure decreased significantly Δ0.20 ± 0.04 MPa *p* = 0.044. Performing the OW-HTO in 5° correction angle resulted in significant decreased contact pressure (Δ0.25 ± 0.10 MPa, *p* = 0.0036) and peak contact pressure (Δ0.39 ± 0.38 MPa, *p* = 0.029) compared with the native test cycle. With a 10° correction angle, OW-HTO significantly decreased area contact pressure by Δ0.32 ± 0.09 MPa, *p* = 0.006 and peak contact pressure by Δ0.48 ± 0.12 MPa, *p* = 0.0654 compared to OW-HTO 5°. Surgical treatment did not result in kinematic changes regarding the superior–inferior translation of the medial joint section. A significant difference was observed for the translation towards the lateral compartment for the ATLAS system Δ1.31 ± 0.54 MPa *p* = 0.022 and the osteotomy Δ3.51 ± 0.92 MPa *p* = 0.001. Furthermore, significant shifting varus to valgus rotation of the treated knee joint was verified for HTO 5° about Δ2.97–3.69° and for HTO 10° Δ4.11–5.23° (pHTO 5 = 0.0012; pHTO 10 = 0.0007) over the entire extension cycle.

**Conclusion:**

OW-HTO results in a significant unloading of the medial compartment. Implantation of an extra-capsular absorbing device did not result in a significant unloading until the implantation technique was applied against the manufacturer’s recommendation. While the clinical difficulty for young and active patients with straight-leg axis and early degenerative changes of the medial compartment persists further biomechanical research to develop sufficient unloading devices is required.

## Introduction

The treatment for symptomatic patients with varus malalignment and consecutively medial compartment osteoarthritis by arranging a straight-leg axis or a discrete overcorrection with the high tibial osteotomy (OW-HTO) is widely accepted [7, 11, 18]. The main goal of this procedure is to shift the stress load from the affected medial compartment, by reorienting the mechanical leg axis towards the lateral sector of the knee. Valgus open-wedge high tibial osteotomy (OW-HTO) is a well-established treatment option in patients with medial compartment osteoarthritis and varus malalignment. Since the introduction of a standardized surgical technique [18], locking plate fixators [19], computer navigation [24], OW-HTO can be considered as a safe procedure with good clinical results [11, 24, 30]. Whereas the clinical benefits of OW-HTO are well documented in the literature, only a few experimental studies have investigated its effect on intra-articular load distribution. A previous study has demonstrated the effective pressure distribution by shifting the loading axis in the very same experimental setup regarding OW-HTO and the Kinespring absorber [8].

While OW-HTO is geared to address the symptomatic patient with varus malalignment, the absorber concept is designed to treat the numerous young, active patients with pronounced degenerative changes of the medial compartment of the knee (e.g., after meniscectomy) with a straight-leg axis [5, 9, 22]. Treatment of these patients remains challenging because neither OW-HTO nor unicompartmental knee replacement seems to be appropriate [27, 29]. As significant clinical benefit in such cases could be achieved by temporary external distraction [18], newly developed extra-capsular absorbing devices, allowing full range of motion, tend to unload the medial compartment in a comparable manner to OW-HTO.

These unloading devices manage to decrease the weight bearing up to 13 kg without substantial modifications of the bony anatomy [8, 10]. The precursor of the ATLAS System (Kinespring System, Moximed, Hayward, CA, USA) proved its unloading potential of the medial compartment in recent studies [8].

First patients reported soft tissue irritation and impingement caused by the implant. Furthermore, the surgical technique was challenging. Therefore, a smaller implant with a facilitated surgical technique was developed. Data proving the unloading effect and its influence on the joint kinematic during physiological gait cycle are missing.

Purpose of the present study was to examine the unloading effect and the consequences for knee kinematics under physiological load in a complete gait cycle.

The hypothesis was that the ATLAS System would provide an unloading effect similar to that achieved with OW-HTO, especially during the stance phase and full extension.

Furthermore, this study illuminates the changes in kinematics and weight bearing after pressure-relief surgery to the medial compartment. OW-HTO results in significant 3D changes of the tibia [16]. Physicians have to consider higher degrees of tibia torsion which might influence overall gait mechanics and specifically alternate the patellofemoral joint [13].

Thus, the purpose of our study was to verify this concomitant change after OW-HTO, respectively, implanting the ATLAS system. The second hypothesis was that the valgus producing OW-HTO does result in translation to the lateral knee sector and intensifying valgus rotation depending on the extent of frontal plane correction.

## Materials and methods

Eight fresh-frozen male knee specimens (mean cadaver age 62.6 ± 4.9 years, weight 76.5 kg ± 26.1) were tested in a specially designed knee simulator that allowed simulation of isokinetic flexion–extension motions under physiological condition. Pairwise testing (four right and left knee specimen) was achieved in all cases. The specimen’s medical history did not show any kind of trauma or previous knee surgery. After thawing the specimens for 24 h at approximately 20 °C, the skin and subcutaneous tissue were removed while preserving the muscles, ligaments, tendons, and joint capsule. The femur and tibia were transected approximately 300 mm proximal and distal to the knee joint line in each specimen and were embedded in barrels using three-component resin in the same manner. Power analysis with a power of 0.8 and *α* of 0.05 indicated that a minimum of seven knees were required for comparison.

### Knee simulator

**Fig. 1:**
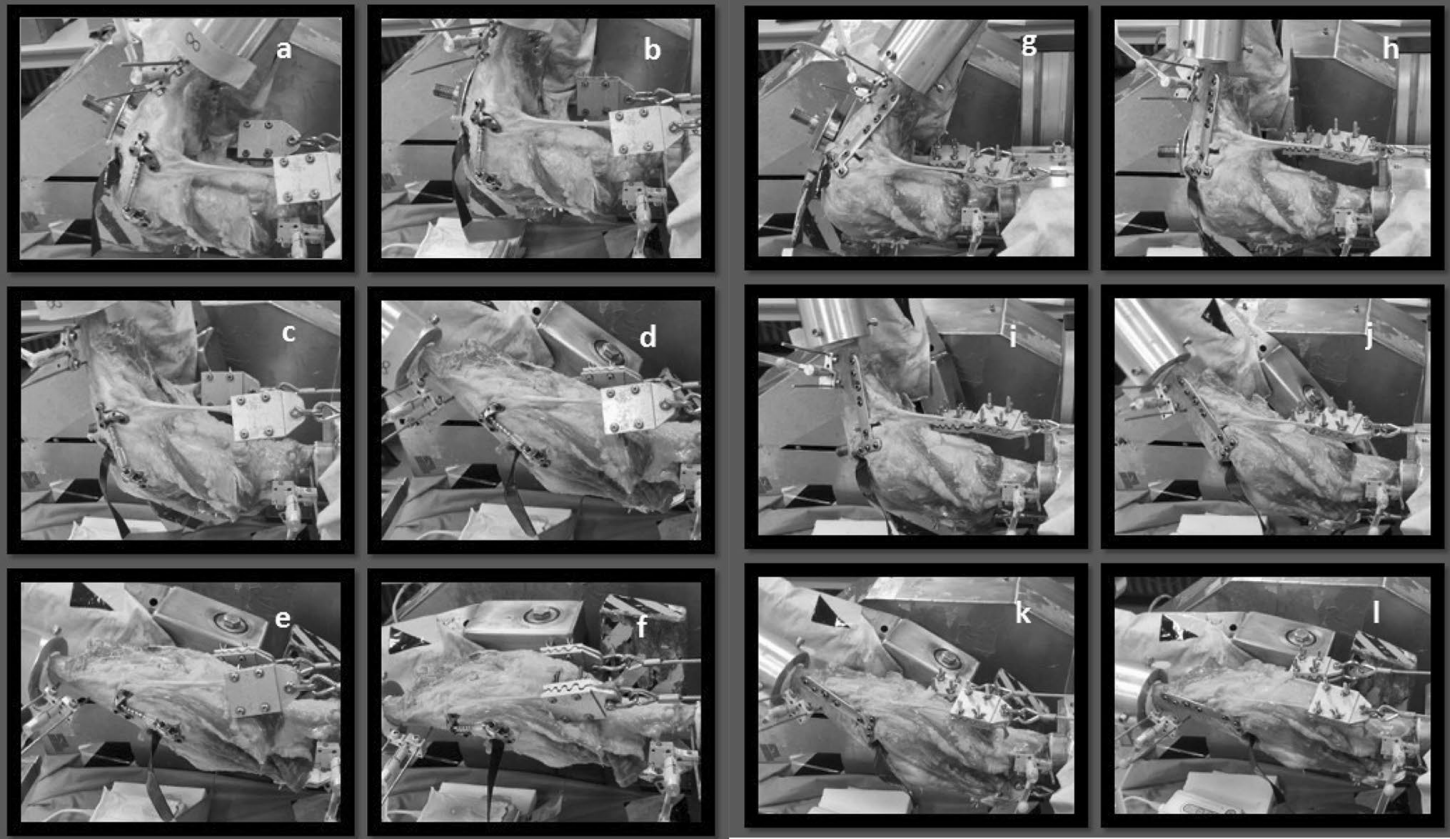
Experimental setting with the knee pointing downward. It is mounted in the biomechanical simulator, which allows isokinetic flexion–extension motion under physiological loading, accomplished by pulling the quadriceps tendon with a defined counterweight at the tibia and hamstrings. **a** Test setting with the knee mounted in 120° flexion showing the absorber (ATLAS) during the test cycle, **b** 90° knee flexion, **c** 75° knee flexion, **d** 45° knee flexion, **e** 15° knee flexion, **f** 0° knee flexion. **g** Test setting with the knee mounted in 120° flexion showing the OW-HTO during the test cycle, **h** 90° knee flexion **i** 75° knee flexion, **j** 45° knee flexion, **k** 15° knee flexion. **l** 0° knee flexion

To simulate isokinetic flexion–extension motions under physiological loading, the knee specimens were mounted into a specially designed knee simulator (that had been used in several previous studies, see Fig. 1) by fixing the femur horizontally and facing the patella downward [1, 8]. The simulation of a physiologic gait cycle is generated with an isokinetic flexion–extension movement under physiological loading. This specific experimental setup was used before and is well established. The tibia was attached at mid-length by means of a linear-rotational bearing, allowing axial sliding and turning as well as rotation transverse to the tibial axis. The bearing itself was attached to a swing arm that permitted varus–valgus rotation. The weight of the swing arm bearing the knee joint was equalized by a counterweight. The load-measuring sensor of the swing arm was self-weight compensated. A strain-gauge-based, load-measuring device was attached to the swing arm, allowing continuous measurement of the tibial extension moment. Three hydraulic cylinders provided movement of the tibia. While the first cylinder simulated variable quadriceps muscle force, the second cylinder simulated constant 100-N flexion force of the hamstrings during the extension cycle; finally, the third cylinder applied an external flexion moment. An isokinetic extension cycle with an angular velocity of 10°/s was performed between 120° knee flexion and full extension using an extension moment of 31 Nm. The flexion angle was measured by an electronic goniometer attached to the swing arm with an accuracy and repeatability of 0.1° at a sampling frequency of 10 Hz.

### Tibiofemoral pressure

Intra-articular pressure in the medial compartment was measured using pressure-sensitive film (K-Scan 4000; Tekscan, Boston, MA, USA) as described previously [2]. The 0.1 mm Teflon film was glued onto the sensors to protect them from shear forces during joint motion.

The pressure films were preconditioned first by five repeated loading and unloading cycles at 3300 N and then calibrated using a two-point method at 800 and 3300 N, which was applied on the entire area of the pressure film in a material testing device (MTS Mini Bionix I; MTS Corporation, Minneapolis, MN, USA) according to the manufacturer’s guidelines.

The knee joint was approached from medial with a parapatellar incision, as well as dorsally by approaching the posterior tibial plateau between musc. semimembranosus and the medial gastrocnemius head. The sensors were attached to the medial compartment by several 1–0 sutures. The area contact pressure (ACP), concerning the entire medial tibiofemoral compartment, and peak contact pressure (PCP) were evaluated.

### Unloading device

A detailed description of the surgical procedure for the ATLAS System implant is quoted elsewhere [26]. The Implantation procedure was performed as recommended by the manufacturer. After the anatomic preparation and attaching the bony ends into the brass tubes fixed with resin, each cadaver knee was locked into position with a vise. In this set-up, we performed the implantation approaching from the medial side, step by step as recommended by the surgical manual and under manufacturer’s supervision. The surgery was conducted with an experienced assistant who could perform different flexion/extension moments as well as Varus-stress if needed. The success of the executed surgery was evaluated optically after each intervention through several surgeons. The specially designed templates and probe device provide equally a valid verification of the implant-position and individually absorber size. After each surgery, joint motion was assessed in both the anteroposterior and lateral planes to confirm appropriate implantation. Especially observation for compression of the absorber in full extension and under varus stress, without recompression in deep flexion or tissue impingement. To test the knee in its natural state, the modular absorber was uncoupled from the femoral and tibial bases and set aside.

**Fig. 2:**
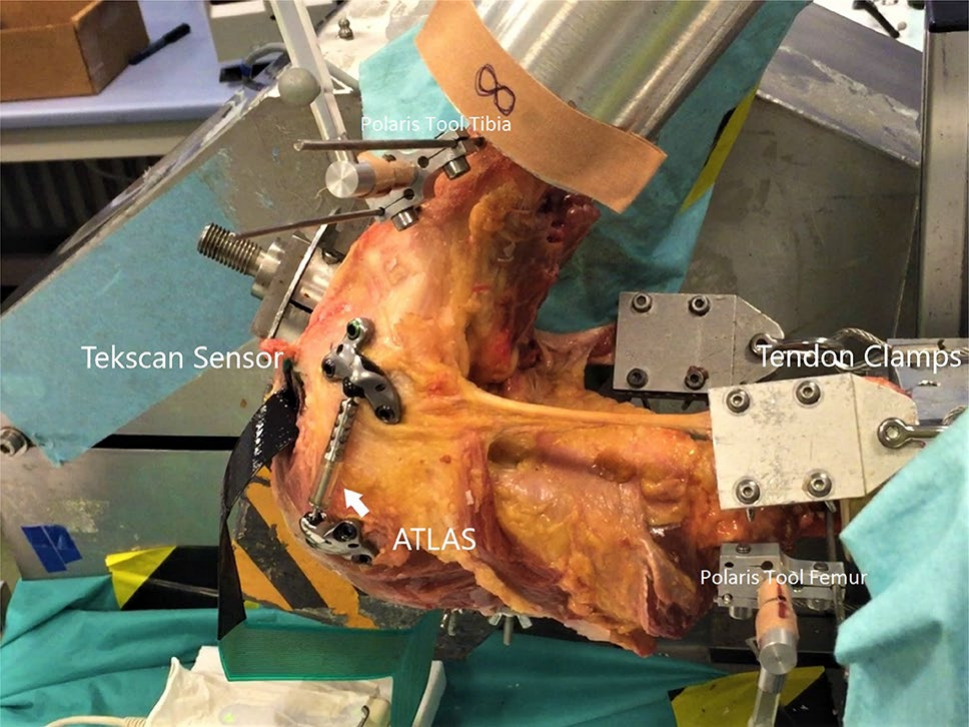
Experimental setting. Mounted knee in kinemator with femoral and tibial base for the 3D tracking passive tools (Polaris). Test cycle with implanted ATLAS system

To test the knee with the ATLAS system, the absorber was once again introduced into the femoral and tibial bases (see Fig. 2). The absorber and bases were removed from the knee before realizing the OW-HTO and fixation with the Tomofix plate (Synthes GmbH, Zuchwil, Switzerland).

### Osteotomies

The surgical technique was identical in all patients. OW-HTO was performed according to the technique recommended by the AO International knee expert group, with at least 50% release of the medial collateral ligament, as recommended [1, 19]. In all cases, a biplanar-step osteotomy, first with a 5° correction angle and later a 10° correction angle, was performed without the use of additional bone grafts. An internal plate fixator was used to stabilize the osteotomy (TomoFix, Solothurn, Synthes, Switzerland).

### 3-D tracking system polaris

The extension cycle was conducted with a measuring system by Polaris (NDI Waterloo Canada). Optical tracking uses a position sensor to detect infrared-emitting or retro-reflective markers affixed to a tool fixated in the bony structures of the embedded knee specimen. The position sensor calculates the position and orientation of the tool based on the information the position sensor receives from those markers. The measuring system records movement in three dimensions with three rotation axis. The superior–inferior, the mediolateral translation and the varus-/valgus rotation were observed. Before each test cycle, the alignment was examined with a passive probe tool and the orientation was matched to the first native test cycle.

### IRB approval

The ethics committee of Hannover University approved this study (ID 3083-2016).

### Statistical analysis

SPSS for Windows (version 25.0; SPSS, Chicago, IL, USA) was used for statistical analyses designed to examine the data in this study. Quantitative variables at baseline were expressed as means ± SD. For statistical evaluation, a pair-sampled *t* test was used. A value of *p* ≤ 0.05 was considered to indicate statistical significance. Power analysis with a power of 0.8 (effect size 0.34) and *α* of 0.05 indicated that a minimum of seven knees were required for comparison.

### Results

- ACP—contact pressure

The mean ACP peaked at 100–115° flexion (1.17–1.27 MPa), decreased at around 90° flexion and increased again from 45° to full extension.

#### ATLAS

Considering the entire cycle of movement from 120 to 0°, implantation of the ATLAS System led to a slightly reduction in ACP from around 80° flexion angle to full extension (mean difference to native test cycle 4–16%). The given samples with a 15° flexion angle interval could not demonstrate significance. There was no significant unloading in throughout the extension cycle.

#### OW-HTO

The implantation of the unloading implant resulted in decreased mean medial compartment ACP values compared with the first test cycle (detailed numbers are given in Table 1). A noticeable significant decrease in pressure values was observed in the osteotomy group (HTO 10° *p* = 0.0006; HTO 5° *p* = 0.0026).

**Table 1:** Mean contact pressure values of the tibiofemoral compartment in detail

Angle	Contact pressure	Mean value	SDS	Min	Max	Mean Diff. To Nativ	*P* value *t* test		
							N vs X	A vs HTO	HTO 5 vs HTO 10
0°	N	0.91	0.30	0.50	1.43				
	A	0.87	0.22	0.63	1.26	4.10%	n.s		
	A + VS	0.77	0.29	0.51	1.30	16.08%	n.s		
	HTO 5	0.77	0.30	0.48	1.45	16.08%	n.s	n.s	
	HTO 10	0.69	0.26	0.34	1.17	24.51%	n.s	n.s	n.s
15°	N	0.74	0.25	0.43	1.09				
	A	0.62	0.22	0.41	1.08	16.28%	n.s		
	A + VS	0.59	0.19	0.33	0.79	20.23%	n.s		
	HTO 5	0.59	0.18	0.33	0.86	20.23%	n.s	n.s	
	HTO 10	0.57	0.12	0.34	0.69	23.07%	n.s	n.s	n.s
30°	N	0.71	0.20	0.39	0.94				
	A	0.66	0.22	0.37	1.03	6.79%	n.s		
	A + VS	0.56	0.17	0.41	0.67	21.12%	n.s		
	HTO 5	0.50	0.14	0.23	0.65	28.93%	0.0469*	n.s	
	HTO 10	0.55	0.05	0.47	0.60	22.50%	n.s	n.s	n.s
45°	N	0.75	0.14	0.56	1.00				
	A	0.70	0.17	0.42	0.91	6.87%	n.s		
	A + VS	0.59	0.11	0.38	0.71	21.32%	0.047		
	HTO 5	0.50	0.10	0.36	0.63	33.20%	0.0036*	0.0031*	
	HTO 10	0.56	0.08	0.40	0.67	25.89%	0.0112*	0.0094*	n.s
60°	N	0.88	0.17	0.56	1.15				
	A	0.78	0.15	0.55	0.96	12.20%	n.s		
	A + VS	0.68	0.14	0.44	0.85	22.75%	0.044		
	HTO 5	0.57	0.13	0.42	0.80	35.29%	0.0026*	0.0121*	
	HTO 10	0.56	0.07	0.47	0.70	36.39%	0.0006*	0.0040*	n.s
75°	N	1.06	0.18	0.78	1.38				
	A	1.04	0.19	0.66	1.35	2.06%	n.s		
	A + VS	0.86	0.17	0.59	0.99	18.87%	n.s		
	HTO 5	0.80	0.11	0.61	0.95	24.37%	0.0101*	0.0135*	
	HTO 10	0.68	0.13	0.46	0.87	36.01%	0.0011*	0.0016*	n.s
90°	N	1.17	0.16	0.92	1.38				
	A	1.21	0.23	0.78	1.59	− 3.42%	n.s		
	A + VS	1.02	0.30	0.61	1.55	12.82%	n.s		
	HTO 5	1.08	0.31	0.54	1.61	7.28%	n.s	n.s	
	HTO 10	1.01	0.34	0.47	1.58	13.59%	n.s	n.s	n.s
105°	N	1.27	0.37	0.32	1.57				
	A	1.30	0.39	0.31	1.61	− 2.74%	n.s		
	A + VS	1.28	0.38	0.32	1.47	− 0.79%	n.s		
	HTO 5	1.10	0.38	0.31	1.42	13.23%	n.s	n.s	
	HTO 10	1.06	0.37	0.31	1.44	16.46%	n.s	n.s	n.s
120°	N	0.51	0.30	0.00	0.90				
	A	0.55	0.15	0.30	0.79	− 7.67%	n.s		
	A + VS	0.52	0.18	0.29	1.01	− 1.96%	n.s		
	HTO 5	0.62	0.28	0.30	1.08	− 22.68%	n.s	n.s	
	HTO 10	0.53	0.25	0.31	1.08	− 4.08%	n.s	n.s	n.s

*N1* native, *A* ATLAS, *A+VS* ATLAS implantation under varus stress, *HTO* high tibial osteotomy with 5- and 10-degrees correction angle

The unloading behavior of the osteotomy showed significant unloading to the native test cycle around flexion angles between 75 and 30° (Table 1). Overall through the whole extension cycle we can recognize constant lower median contact pressure values. The percentage decrease ranges from 16 to 38%. An increase in the correction angle to 10° also resulted in greater unloading relative to that achieved using the absorber and 5° OW-HTO (Table 1).

b) PCP—peak pressure

The mean PCP peaked similar to ACP at 90–100° flexion (2.29–2.30 MPa), decreased at around 80–60° flexion, and increased again from 30° to full extension.

#### ATLAS

Considering the entire cycle of movement from 120 to 0°, implantation of the ATLAS System led to a slightly reduction in PCP from around 75° flexion angle to full extension (mean difference to native test cycle 3–19%).

The implantation of the unloading implant resulted in decreased mean peak pressure values compared with the native test cycle (detailed numbers are given in Table 2). The ATLAS System could not demonstrate significance.

**Table 2:** Mean peak pressure values of the tibiofemoral compartment in detail

Angle	Peak pressure	Mean value	SDS	Min	Max	Mean Diff. To Nativ	*P* value *t* test		
							N vs X	A vs HTO	HTO 5 vs HTO 10
0°	N	1.95	0.59	0.76	2.78				
	A	1.87	0.46	1.17	2.53	4.31%	n.s		
	A + VS	1.87	0.44	1.19	2.50	4.31%	n.s		
	HTO 5	1.56	0.78	0.70	3.31	19.89%	n.s	n.s	
	HTO 10	1.28	0.55	0.70	2.19	34.21%	n.s	n.s	n.s
15°	N	1.49	0.72	0.57	2.41				
	A	1.19	0.55	0.56	2.00	19.56%	n.s		
	A + VS	1.21	0.57	0.55	2.11	18.71%	n.s		
	HTO 5	1.17	0.51	0.47	1.82	21.15%	n.s	n.s	
	HTO 10	0.98	0.34	0.40	0.69	33.84%	n.s	n.s	n.s
30°	N	1.39	0.58	0.65	2.32				
	A	1.30	0.58	0.55	2.45	6.39%	n.s		
	A + VS	1.15	0.53	0.35	2.11	10.10%	n.s		
	HTO 5	0.94	0.46	0.25	1.79	32.15%	n.s	n.s	
	HTO 10	0.97	0.29	0.67	1.56	30.61%	n.s	n.s	n.s
45°	N	1.49	0.36	1.03	2.07				
	A	1.42	0.59	0.46	2.29	5.19%	n.s		
	A + VS	1.28	0.47	0.44	1.92	12.75%	n.s		
	HTO 5	1.10	0.39	0.51	1.72	26.23%	0.0299*	0.0314*	
	HTO 10	1.15	0.43	0.45	1.88	23.11%	n.s	n.s	n.s
60°	N	1.68	0.49	0.89	2.45				
	A	1.52	0.58	0.84	2.52	9.33%	n.s		
	A + VS	1.35	0.46	0.71	2.22	19.65%	n.s		
	HTO 5	1.17	0.39	0.59	1.67	30.46%	n.s	n.s	
	HTO 10	1.20	0.37	0.69	1.84	28.71%	n.s	n.s	n.s
75°	N	2.11	0.49	1.26	2.77				
	A	2.02	0.46	1.01	2.67	3.91%	n.s		
	A + VS	1.78	0.42	0.93	2.47	15.64%	n.s		
	HTO 5	1.93	0.49	1.04	2.75	8.28%	n.s	n.s	
	HTO 10	1.63	0.41	0.89	2.11	22.48%	n.s	n.s	n.s
90°	N	2.29	0.33	1.78	2.86				
	A	2.37	0.49	1.73	3.22	− 3.72%	n.s		
	A + VS	2.32	0.99	1.11	3.84	− 1.31%	n.s		
	HTO 5	2.53	1.35	1.04	5.45	− 10.57%	n.s	n.s	
	HTO 10	2.38	1.07	1.12	4.63	− 4.14%	n.s	n.s	n.s
105°	N	2.30	0.74	0.44	2.85				
	A	2.39	0.83	0.37	3.08	− 3.73%	n.s		
	A + VS	2.32	0.91	0.41	3.01	− 0.87%			
	HTO 5	2.20	0.90	0.37	3.16	4.46%	n.s	n.s	
	HTO 10	2.06	0.80	0.37	3.06	10.42%	n.s	n.s	n.s
120°	N	0.77	0.54	0.00	1.52				
	A	0.76	0.29	0.30	1.11	0.93%	n.s		
	A + VS	0.82	0.51	0.34	1.06	− 6.49%	n.s		
	HTO 5	0.93	0.55	0.30	1.93	− 21.25%	n.s	n.s	
	HTO 10	0.86	0.56	0.31	1.96	− 11.98%	n.s	n.s	n.s

*N1* native, *A* ATLAS, *A+VS* ATLAS implantation under varus stress, *HTO* high tibial osteotomy with 5- and 10-degrees correction angle

#### OW-HTO

A noticeable significant decrease in pressure values was observed in the osteotomy group (HTO 5° *p* = 0.0029 PCP). Throughout the whole extension cycle the osteotomy group (HTO 5/10) showed percentage decrease in peak pressure values (HTO 5 = 8–30%; HTO 10 = 22–34%).

The testing set up allowed also visualizing the kinematic changes in the knee joint following surgical intervention.

c) Superior–inferior translation

Surgical treatment of the native knee did not result in kinematic changes for the superior–inferior translation of the tibia plateau (Table 3).

**Table 3:** Tibia—femur motion: superior–inferior translation (absolute in mm)

Angle	Test cycle	Mean value	SDS	Min	Max	Mean Diff. To Nativ	*P* value *t* test		
							N vs X	A vs HTO	HTO 5 vs HTO 10
0°	N	− 29.57	15.45	− 45.97	− 1.67				
	A	− 30.0	14.84	− 45.79	− 2.57	− 1.45%	n.s		
	HTO 5	− 33.34	16.95	− 48.29	− 0.69	− 12.7%	n.s	n.s	
	HTO 10	− 33.99	17.54	− 49.57	− 0.33	− 14.9%	n.s	n.s	n.s
15°	N	− 30.05	15.10	− 48.37	− 2.07				
	A	− 30.54	14.86	− 48.44	− 2.57	− 1.63%	n.s		
	HTO 5	− 33.06	17.19	− 49.79	− 0.53	− 10.0%	n.s	n.s	
	HTO 10	− 33.59	17.60	− 51.20	− 0.39	− 11.7%	n.s	n.s	n.s
30°	N	− 29.89	15.96	− 49.14	0.16				
	A	− 30.12	16.00	− 49.66	0.17	− 0.77%	n.s		
	HTO 5	− 31.82	17.71	− 50.98	− 0.30	− 6.05%	n.s	n.s	
	HTO 10	− 32.20	18.11	− 52.61	0.04	− 7.72%	n.s	n.s	n.s
45°	N	− 27.11	17.27	− 50.19	4.50				
	A	− 26.96	17.65	− 50.21	5.26	0.55%	n.s		
	HTO 5	− 27.56	18.87	− 50.92	3.48	− 1.66%	n.s	n.s	
	HTO 10	− 28.16	19.34	− 51.67	3.47	− 3.87%	n.s	n.s	n.s
60°	N	− 22.29	18.70	− 51.21	9.97				
	A	− 22.16	19.07	− 50.96	10.76	0.59%	n.s		
	HTO 5	− 21.31	20.09	− 49.62	8.79	4.40%	n.s	n.s	
	HTO 10	− 21.48	20.52	− 49.94	8.92	3.63%	n.s	n.s	n.s
75°	N	− 16.93	19.81	− 49.42	15.21				
	A	− 16.46	19.96	− 48.94	15.98	2.77%	n.s		
	HTO 5	− 14.75	21.11	− 47.27	14.13	12.87%	n.s	n.s	
	HTO 10	− 14.46	21.18	− 46.47	13.70	14,54%	n.s	n.s	n.s
90°	N	− 10.87	20.23	− 45.54	20.16				
	A	− 10.32	20.54	− 44.76	21.07	5.06%	n.s		
	HTO 5	− 7.47	21.35	− 41.99	19.14	31.34%	n.s	n.s	
	HTO 10	− 7.03	21.59	− 41.59	18.74	35.35%	n.s	n.s	n.s
105°	N	− 4.47	20.11	− 39.23	26.15				
	A	− 3.72	20.51	− 38.51	27.33	16.84%	n.s		
	HTO 5	− 0.13	21.28	− 34.57	26.12	97.12%	n.s	n.s	
	HTO 10	0.59	21.59	− 34.29	26.07	113.91%	n.s	n.s	n.s
120°	N	− 0.08	19.22	− 32.38	31.54				
	A	1.04	19.77	− 31.79	32.77	96.22%	n.s		
	HTO 5	4.33	20.31	− 28.48	31.68	−	n.s	n.s	
	HTO 10	5.12	20.15	− 26.94	31.32	−	n.s	n.s	n.s

*N1* native, *A* ATLAS, *HTO* high tibial osteotomy, with 5- and 10-degrees correction angle

#### ATLAS/ OW-HTO

Throughout the extension cycle neither the ATLAS nor the OW-HTO affected the translation in the sagittal plane for superior–inferior shifting of the medial tibial plateau.

d) Medio-lateral translation

Significant difference was observed for the translation towards the lateral compartment (Table 4) after surgery. Both procedures demonstrated shifting for the tibia axis towards the lateral compartment of the tibiofemoral knee joint.

**Table 4:** Tibia—femur motion: mediolateral translation (absolute in mm)

Angle	Test cycle	Mean value	SDS	Min	Max	Mean Diff. To Nativ	*P* value *t* test		
							N vs X	A vs HTO	HTO 5 vs HTO 10
0°	N	3.74	5.68	− 4.69	11.92				
	A	4.73	6.07	− 5.29	13.63	− 26.47%	0.047	n.s	n.s
	HTO 5	7.35	14.39	− 8.33	31.95	− 96.52%	0.004	n.s	n.s
	HTO 10	6.67	14.25	− 9.86	29.41	− 78,34%	0.010	n.s	n.s
15°	N	2.57	4.32	− 3.69	11.11				
	A	3.52	5.34	− 3.85	12.20	− 36,96%	0.041	n.s	n.s
	HTO 5	6.42	14.58	− 8.13	32.72	− 149,80%	0.008	n.s	n.s
	HTO 10	5.99	14.21	− 8.12	30.07	− 133.07%	0.019	n.s	n.s
30°	N	1.77	4.25	− 4.42	10.19				
	A	2.77	5.47	− 4.47	11.17	− 56.49%	0.022	n.s	n.s
	HTO 5	5.59	14.57	− 8.80	33.19	− 215.81%	0.007	n.s	n.s
	HTO 10	5.22	14.26	− 8.96	30.99	− 194.91%	0.009	n.s	n.s
45°	N	2.16	4.05	− 2.58	9.86				
	A	3.06	5.30	− 3.01	11.61	− 41.66%	0.039	n.s	n.s
	HTO 5	5.45	14.57	− 8.44	33.68	− 152.31%	0.005	n.s	n.s
	HTO 10	5.08	13.80	− 7.95	30.55	− 135.18%	0.010	n.s	n.s
60°	N	2.04	4.21	− 3.13	8.92				
	A	3.05	5.66	− 3.23	13.75	− 49.05%	0.031	n.s	n.s
	HTO 5	5.07	14.34	− 8.84	33.37	− 148.52%	0.006	n.s	n.s
	HTO 10	4.72	13.68	− 8.74	30.34	− 131.37%	0.009	n.s	n.s
75°	N	1.71	4.35	− 3.50	8.47				
	A	2.62	5.90	− 4.05	14.33	− 53.21%	0.044	n.s	n.s
	HTO 5	4.31	13.56	− 9.03	31.09	− 152.04%	0.013	n.s	n.s
	HTO 10	4.08	13.32	− 9.53	29.35	− 138.59%	0.018	n.s	n.s
90°	N	1.05	4.71	− 4.70	9.43				
	A	1.91	6.08	− 4.93	14.48	− 81.90%	n.s	n.s	n.s
	HTO 5	3.23	13.45	− 9.63	30.47	− 207.61%	0.028	n.s	n.s
	HTO 10	2.99	12.72	− 10.20	27.54	− 184.76%	0.027	n.s	n.s
105°	N	0.40	4.67	− 6.16	8.62				
	A	1.05	6.03	− 6.35	13.66	− 162.50%	n.s	n.s	n.s
	HTO 5	2.62	12.49	− 7.83	28.24	−	n.s	n.s	n.s
	HTO 10	2.05	11.96	− 10.47	25.04	−	n.s	n.s	n.s
120°	N	− 0.13	4.89	− 7.35	7.70				
	A	0.29	6.27	− 7.86	12.95	−	n.s	n.s	n.s
	HTO 5	2.45	12.08	− 6.71	27.41	−	n.s	n.s	n.s
	HTO 10	2.15	10.97	− 6.67	23.99	−	n.s	n.s	n.s

*N1* native, *A* ATLAS, *HTO* high tibial osteotomy with 5- and 10-degrees correction angle

#### ATLAS

Implantation of the ATLAS system showed translation towards lateral in the coronar plane (Δ1.31 mm native—ATLAS) and significantly relevant (*p* = 0.031).

#### OW-HTO

The total amount of translation was much higher in the osteotomy group Δ3.51/Δ3.86 mm (native vs. HTO 5°/HTO 10°) For flexion moments from 0–90° [[p (HTO5°) = 0.004–0.027] [*p* (HTO 10°) = 0.00014–0.027], the OW-HTO displayed significance compared to the native knee.

e) Varus–valgus rotation

Rotation in the coronar plane was observed for both surgical treatments.

#### ATLAS

Significant discrepancy was observed for the osteotomies, the ATLAS system showed a little amount of valgus momentum towards full extension (Δ0.3–0.7 mm native—ATLAS).

#### OW-HTO

The OW-HTO modified the valgus/varus alignment in the coronar plane significantly (Table 5). With greater correction angle, the shifting to valgus rotation increased continual. The effect was observed during the complete extension cycle from 0 to 120°. The valgus rotation angle differed from the untreated knee for HTO 5° about Δ2.97–3.69° and for HTO 10° Δ4.11–5.23° (pHTO 5 = 0.0012; pHTO 10 = 0.0007).

**Table 5:** Tibia—femur motion: varus–valgus rotation (absolute in °)

Angle	Test Cycle	Mean value	SDS	Min	Max	Mean Diff. To Nativ	*P* value *t* test		
							N vs X	A vs HTO	HTO 5 vs HTO 10
0°	N	− 2.94	3.68	− 7.10	4.71				
	A	− 3.48	3.95	− 8.45	4.72	− 18.36%	n.s		
	HTO 5	− 6.68	4.87	− 11.70	2.33	− 127.21%	0.0012	0.045	
	HTO 10	− 7.74	4.72	− 13.25	0.38	− 163.26%	0.0007	0.043	n.s
15°	N	− 4.08	4.65	− 9.27	4.45				
	A	− 4.70	4.99	− 9.71	4.36	− 15.19%	n.s		
	HTO 5	− 7.26	5.35	− 14.46	1.68	− 77.94%	0.0004	0.038	
	HTO 10	− 8.58	5.64	− 16.60	0.06	− 110.29%	0.0001	0.027	n.s
30°	N	− 4.85	5.24	− 11.63	2.30				
	A	− 5.33	5.55	− 12.41	2.43	− 9.89%	n.s		
	HTO 5	− 7.43	5.75	− 16.39	0.76	− 53.19%	0.005	n.s	
	HTO 10	− 8.69	6.38	− 18.40	− 0.34	− 79.38%	0.0017	n.s	n.s
45°	N	− 4.98	6.00	− 12.88	1.61				
	A	− 5.32	6.20	− 13.50	1.25	− 6.82%	n.s		
	HTO 5	− 7.37	6.45	− 17.38	− 0.51	− 47.99%	0.008	n.s	
	HTO 10	− 8.48	7.10	− 19.43	− 0.68	− 70.82%	0.002	n.s	n.s
60°	N	− 4.59	6.48	− 13.12	3.31				
	A	− 4.86	6.55	− 13.28	2.82	− 5.88%	n.s		
	HTO 5	− 7.06	7.23	− 17.83	1.10	− 53.81%	0.012	n.s	
	HTO 10	− 8.02	8.03	− 19.97	0.86	− 74.72%	0.009	n.s	n.s
75°	N	− 3.84	6.83	− 13.28	4.90				
	A	− 4.00	6.79	− 13.35	4.37	− 4.16%	n.s		
	HTO 5	− 6.45	8.06	− 17.84	3.20	− 67.96%	0.011	n.s	n.s
	HTO 10	− 7.68	8.84	− 20.05	2.83	− 100.02%	0.007	n.s	n.s
90°	N	− 2.53	6.60	− 12.02	6.31				
	A	− 2.67	6.53	− 11.99	5.83	− 5.53%	n.s		
	HTO 5	− 5.34	8.54	− 16.69	5.58	− 111.06%	0.02	n.s	
	HTO 10	− 6.60	9.41	− 19.17	5.41	− 160.86%	0.017	n.s	n.s
105°	N	− 0.64	6.42	− 9.48	7.55				
	A	− 0.57	6.32	− 9.57	7.40	10,93%	n.s		
	HTO 5	− 4.00	9.10	− 14.32	8.30	−	0.0004	0.003	
	HTO 10	− 5.04	9.87	− 16.97	8.15	−	0.00014	0.0002	n.s
120°	N	0.05	6.76	− 8.69	7.05				
	A	0.31	6.30	− 7.64	6.98	−	n.s		
	HTO 5	− 3.63	9.46	− 15.36	9.27	−	0.024	0.031	
	HTO 10	− 4.95	10.37	− 16.62	8.98	−	0.008	0.016	n.s

*N1* native, *A* ATLAS, *HTO* high tibial osteotomy with 5- and 10-degrees correction angle

The detailed results are shown in Tables 1, 2, 3, 4 and 5.

An additional test cycle with implantation of the ATLAS system while applying varus stress during the implantation of the fixed bases and using a larger spring size was performed. This modified mounting revealed significant pressure diminution. Contact pressure decreased significantly Δ0.20 ± 0.04 MPa *p* = 0.044 after deviation from the surgery manual provided by the manufacturer (see Tables 1and 2 A+VS).

## Discussion

The major finding of the present study was that implantation of an extra-articular extra-capsular absorber (ATLAS System) did not achieve an unloading effect on the medial compartment until adaption of the surgical technique by applying varus stress during implantation and installation of a sample size larger than measured with the measuring device supplied by the manufacturer. Applying varus stress while positioning, the distance between the femoral and tibial sockets was reduced and therefore the potency of the absorber augmented. See Tables 1 and 2 (A+VS).

Consequently, the surgical technique as well as the measuring device needs to be adapted by the manufacturer if sufficient unloading of the medial compartment shall be achieved.

Unloading the medial compartment is an essential treatment for medial osteoarthritis of the knee [1, 15]. In a clinical study, joint distraction allowed cartilage regeneration, with significant pain relief even at 1 year postoperatively [14]. The introduced treatments in this study pursue this objective. Valgus OW-HTO in patients with varus OA has previously demonstrated positive effects on the clinical outcome [6, 11].

Young and active patients with medial OA but a straight-leg axis are challenging as OW-HTO is contraindicated in patients without malalignment, and unicondylar knee replacement is related to a high risk of failure owing to the young age and high activity level of the patients [28].

The basic idea of the unloading device investigated in the present study is to transfer the applied weight during gait cycle directly from the distal femur to the proximal tibia without putting stress on the cartilage of the medial compartment [25]. The effectiveness was not yet examined in biomechanical studies, even though there are several studies on the predecessor [8, 17].

The effect of the precursor (Kinespring) in the very same setup showed significant pressure relief in a static model and a biomechanical model simulating the gait cycle [8].

Similar to the precedent work, the main purpose of the present study was to compare the unloading effect of the ATLAS System and OW-HTO during dynamic full range of motion under physiological conditions. The test setting applied in the present study is well established and has been described previously [1].

Comparable to the study by Bode et al. [8], a significant unloading effect for ACP and PCP, especially near but not at full extension, was observed for OW-HTO. The intra-articular pressures were comparable to those of earlier studies [1, 4, 8, 17]. With respect to range of motion, OW-HTO achieved unloading from 60° of flexion to almost full extension while the absorber did not have a significant effect. In contrast, from 30 flexion to 75° flexion, the HTO (5° and 10°) showed a significant discharging effect. Affirming the conclusion that valgus OW-HTO covers the essential range of weight-bearing during a physiological gait cycle. The biomechanical results are consistent with the previous study and clinical outcomes and underline the positive effect of pressure relief.

Surgery-associated modification to tibial torsion after valgus producing OW-HTO has been examined by several studies [3, 16, 20]. A study by Kendoff et al. describes external rotation of the distal tibial fragment (allover 2.7 ± 6.3°, 12° max external; 9.5° max internal)—matching the results from this study. Cadaveric specimen under in vitro conditions were tested, even though in previous studies the effect of soft tissue tension remains unclear.

Previous studies demonstrated a significant interaction between the alignment of the tibia after OW-HTO and medial tibiofemoral compartment contact pressures [12, 17, 21] which may provoke excessive effects on the kinematics of the patellofemoral joint. To avoid excessive postoperative torsional changes, the simple intraoperative K-wire method enables the surgeon to estimate the rotation of the distal fragment before finally fixating it. Accuracy of correction of alignment is a crucial factor in determining patient outcome. Preoperative planning and intraoperative navigation are helpful tools to reach patients satisfaction. A meta-analysis showed that the use of navigation in OW-HTO could improve accuracy in both coronal and sagittal alignments but its clinical benefit is still unclear [23, 24].

The present study revealed a significant progress of valgus rotation after OW-HTO, while the ATLAS absorber did not manipulate the rotation-axis. Both techniques presented a shift towards the lateral compartment which is coherent to the pressure distribution on the tibiofemoral joint. Thus, especially the OW-HTO fulfills the proposition to relief pressure to the medial contact area of the medial compartment. The ATLAS system has a surgical limitation, but the modified setup accomplished similar results.

## Limitations

This study has several limitations. Cadaveric biomechanical models only approximate in vivo conditions. The sensitive sensors for measuring pressure during the test cycle were used for four, respectively, five consecutive cycles. Their disposition to crinkling, temperature exposition or malpositioning while testing, although fixed by multiple sutures can advisedly affect the measuring accuracy. Therefore, all sensors were protected with a Teflon cover prior to calibration, and their functionality was evaluated after each test cycle.

The knee simulator was missing a weight-bearing component and can also just approximate the human gait under physiological conditions. Divergent from other in vitro investigations, physiological muscle forces were applied by attaching extensor and flexor muscles by tendon clamps. Additionally, knee kinematics in the setting used in this study was shown to be representative of physiological conditions in human knee joints.

Evaluation of the mechanical and anatomical leg axis of the tested knees could not be evaluated due to resection proximal and distally of the knee joint. Thus, OW-HTO in the straight-leg axis knee although contraindicated in clinical practice could have been performed. To diminish this risk, all knees were examined prior to testing whether intra-articular structures showed signs of degeneration or malalignment.

The measuring system supported the position preservation of the treated knee after surgery and reinstalling in the kinemator. The passive tools fixed to the patella, femur and tibia were recalibrated and matched with the linear probe tool, while identifying the anatomical landmarks. With the help of a reference coordinate system angle—correlations could be balanced to recreate the same starting point and exact flexion angle. This very important step was launched before each test cycle to allow reproducibility.

In summary, the present biomechanical in vitro study compares the unloading potential of the medial compartment under physiological conditions using a medial open-wedge osteotomy versus the successor model of the previously tested extra-articular, extra-capsular absorber (ATLAS). Young, active patients with a straight-leg axis and unicompartmental osteoarthritis currently face a therapeutic gap. Unloading the medial compartment using an extra-capsular absorber might be an option to fill this gap without compromising the bony structure to interfere with following surgery.

While the manufacturer did decide not to distribute the tested device any longer, mainly due to results achieved in the present study, the underlying idea of extra-capsular extra-articular absorbing device should be followed up a matter as this specific patient cohort is definitely a challenge to every knee surgeon without satisfying therapeutical options.

## Conclusion

Implantation of an extra-articular, extra-capsular absorber may lead to unloading in the medial compartment if varus stress is applied during implantation and the absorbing device offers sufficient distraction of the medial compartment.

While OW-HTO once again proved its capability to unload the medial compartment significantly by shifting the weight bearing axis, extra-capsular, extra-articular absorbing devices do require further improvement of the implant as well as the surgical technique.
